# Patient-specific colorectal-cancer-associated fibroblasts modulate tumor microenvironment mechanics

**DOI:** 10.1016/j.isci.2024.110060

**Published:** 2024-05-21

**Authors:** Auxtine Micalet, Anuja Upadhyay, Yousef Javanmardi, Camila Gabriela de Brito, Emad Moeendarbary, Umber Cheema

**Affiliations:** 1 UCL Centre for 3D Models of Health and Disease, Department of Targeted Intervention, Division of Surgery and Interventional Science, University College London, Charles Bell House, 43-45 Foley Street, London W1W 7TS, UK; 2 Department of Mechanical Engineering, University College London, Gower Street, London WC1E 6BT, UK; 3 Department of Cellular Pathology, Royal Free Hospital, Pond St, London NW3 2QG, UK; 4 199 Biotechnologies Ltd, Gloucester Road, London W2 6LD, UK

**Keywords:** cell biology, stem cells research, biomechanics, cancer

## Abstract

Cancer-associated fibroblasts (CAFs) play a major role in reorganizing the physical tumor micro-environment and changing tissue stiffness. Herein, using an engineered three-dimensional (3D) model that mimics the tumor’s native biomechanical environment, we characterized the changes in matrix stiffness caused by six patient-specific colorectal CAF populations. After 21 days of culture, atomic force microscopy (AFM) was performed to precisely measure the local changes in tissue stiffness. Each CAF population exhibited heterogeneity in remodeling capabilities, with some patient-derived cells stiffening the matrix and others softening it. Tissue stiffening was mainly attributed to active contraction of the matrix by the cells, whereas the softening was due to enzymatic activity of matrix-cleaving proteins. This measured heterogeneity was lost when the CAFs were cocultured with colorectal cancer cells, as all samples significantly soften the tissue. The interplay between cancer cells and CAFs was critical as it altered any heterogeneity exhibited by CAFs alone.

## Highlights

• Patient-specific CAFs exhibited heterogeneity in tissue remodeling capabilities
• Tissue stiffening was mainly attributed to the contraction of the matrix by cells
• Tissue softening was due to enzymatic activity of matrix-cleaving proteins
• Interplay between cancer cells and CAFs was critical as it stopped heterogeneity

## Graphical abstract

**Figure:**
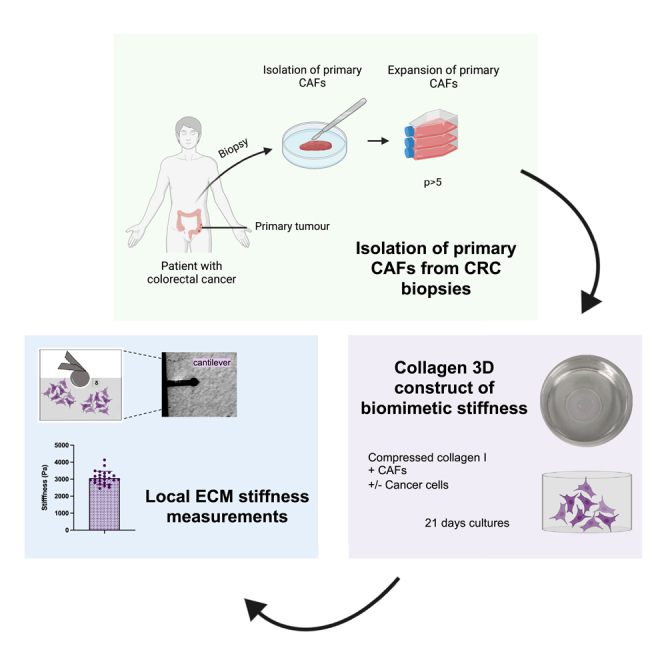

## Introduction

The influence of tissue architecture, and stiffness, on tumor initiation and progression is well recognized.^1^^,^^2^^,^^3^^,^^4^^,^^5^^,^^6^^,^^7^^,^^8^ The tumor micro-environment (TME) is characterized by a changed ECM stiffness that is dictated by fiber deposition, fiber cross-linking,^9^^,^^10^^,^^11^^,^^12^^,^^13^ fiber re-alignment,^14^ matrix contraction,^7^^,^^15^^,^^16^^,^^17^^,^^18^ and local enzymatic matrix degradation.^19^^,^^20^^,^^21^ Those changes are known to induce epithelial-to-mesenchymal transition (EMT) and promote cancer cell invasiveness, proliferation, and overall aggressiveness.^4^^,^^22^^,^^23^^,^^24^^,^^25^ Cancer cells have been shown to both indirectly and actively participate in the changes in matrix stiffness.^2^^,^^19^ However, they do not drive matrix remodeling alone. They recruit non-malignant stromal cells and induce a change in their phenotype, rendering them cancer-associated stromal cells (CASCs).^26^^,^^27^ The recruited stromal cells range in type and include vascular endothelial cells, adipocytes, fibroblasts, organ-specific stromal cells, and mesenchymal stem cells.

Cancer-associated fibroblasts (CAFs) are of particular interest as fibroblasts are responsible for ECM maintenance, deposition, and reabsorption. CAFs are thought to be the main driver of the desmoplastic reaction in cancer, via intense collagen deposition and matrix re-alignment.^26^^,^^28^^,^^29^^,^^30^ They have been shown to lead the way of cancer invasion by creating tracks of rearranged matrix for the cancer cells to follow.^31^ Perpendicularly arranged collagen fibers are linked to increased invasion, as they allow the cells to migrate more efficiently along them.^14^ They secrete key matrix remodeling factors such as transforming growth factor β (TGF-β), lysyl oxidase (LOX), and matrix metalloproteinases (MMPs).^29^

Common CAF markers are α-smooth muscle actin (αSMA), vimentin, PDGFRα, PDGFRβ, and fibroblast activation protein α (FAP), although the CAF population has been shown to be highly heterogeneous and not all markers need to be expressed for the cells to be considered “CAFs.”^30^^,^^32^ Though the majority of CAFs appear to originate from the tissue’s native fibroblasts, it has been shown that other cell populations, such as mesenchymal stromal cells, endothelial cells, and mature adipocytes, can give rise to CAFs, explaining the heterogeneity in markers.^29^^,^^32^ This has most recently been shown by single cells transcriptomics of CAFs found in various solid-state tumors, including colorectal cancer.^33^^,^^34^ Luo et al. have been able to identify through the transcriptome a CAF’s origin (e.g., endothelia or macrophage).^34^ The consensus statement “A framework for advancing our understanding of cancer-associated fibroblasts” by Sahai et al. defines a CAF as “any mesenchymal cell cultured from a tumor that complies with an elongated morphology and is positive for a mesenchymal marker, such as vimentin.”^30^

The aim of this study was to use a three-dimensional (3D) *in vitro* model recapitulating the native biophysical environment to mechanically characterize the changes in TME stiffness caused by patient-specific CAFs alone or in combination with colorectal cancer cells.

**Figure 1:**
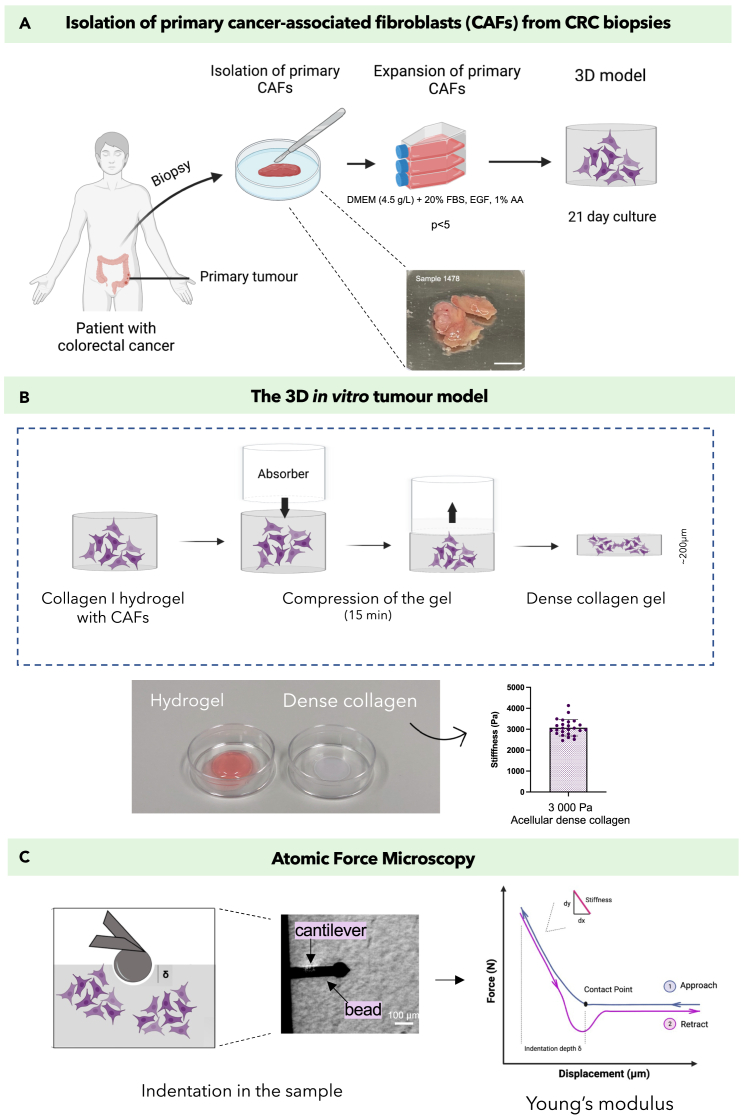
Schematic of experimental methodologies (A) Cancer-associated fibroblasts were isolated from colorectal cancer biopsies and expanded in 2D before embedding them in 3D models of dense collagen matrix. (B) The 3D models were fabricated through plastic compression of collagen I hydrogels seeded with CAFs cells or CAF-cancer cell cocultures. The plastic compression is performed by placing a RAFT absorber on the hydrogel for 15 min, resulting in dense collagen models. The dense collagen 3D models are of biomimetic stiffness (3 kPa). (C) Atomic force microscopy (AFM) was performed on the samples after 21 days of culture, to determine the apparent Young’s modulus (stiffness). Schematics created with BioRender.

Indeed, to study *in vitro* how CAFs remodel their ECM, a highly biomimetic 3D model of physiological relevant ECM composition and stiffness is needed. However, modeling the correct bio-physical properties in *in vitro* models is often overlooked. The 3D model used herein is made of a dense “compressed” collagen I matrix^35^^,^^36^^,^^37^^,^^38^^,^^39^ (Figures 1A and 1B). The dense collagen matrix correctly recapitulate *in vivo* tumor collagen density and stiffness.^2^ Colorectal cancer tissue has been reported to be 1 to 6 kPa,^40^^,^^41^ whereas our model has been measured to be 3kPa^2^ (Figure 1B), which sits perfectly within that real tumor tissue range.

Atomic force microscopy (AFM) was used to precisely measure the local changes in matrix stiffness (Figure 1C). AFM is an instrument that indents the surface of the sample. The indentation depth and applied force is then converted to a resulting Young’s modulus, i.e., stiffness. AFM allows for multiple local measures of stiffness, at the scale where cells sense their microenvironment (the micro-scale), as opposed to rheology where only one single bulk stiffness measurement per sample can be obtained.

We have previously reported on the changes in matrix stiffness caused by highly invasive and less invasive cancer cells,^2^^,^^19^ as well as the impact of patient-specific CAFs on changes in EMT status and invasiveness in cancer cells^42^ using our model of physiological matrix. Here, we aimed to further the investigation and report on the bio-mechanical changes in TME caused by CAFs. To the best of our knowledge, this is the first time that this type of mechanical characterization of patient-derived CAFs, and CAFs/cancer cells, in a highly biomimetic 3D environment has been conducted.

## Results

### Isolation and characterization of patient-specific cancer-associated fibroblasts

Colon tumor biopsies were obtained in limited quantity from the Royal Free hospital, London, UK, from patients undergoing diagnostic surgeries. All biopsies were taken from untreated primary tumors.

From the collected colon tumor biopsies, six patient-derived CAF samples were established and expanded in 2D, up to a maximum of five passages (Figure 1A). Patient information for each sample can be found in Table S1. These data were compiled by a pathologist and reports on information such as TNM staging and cell differentiation.

**Figure 2:**
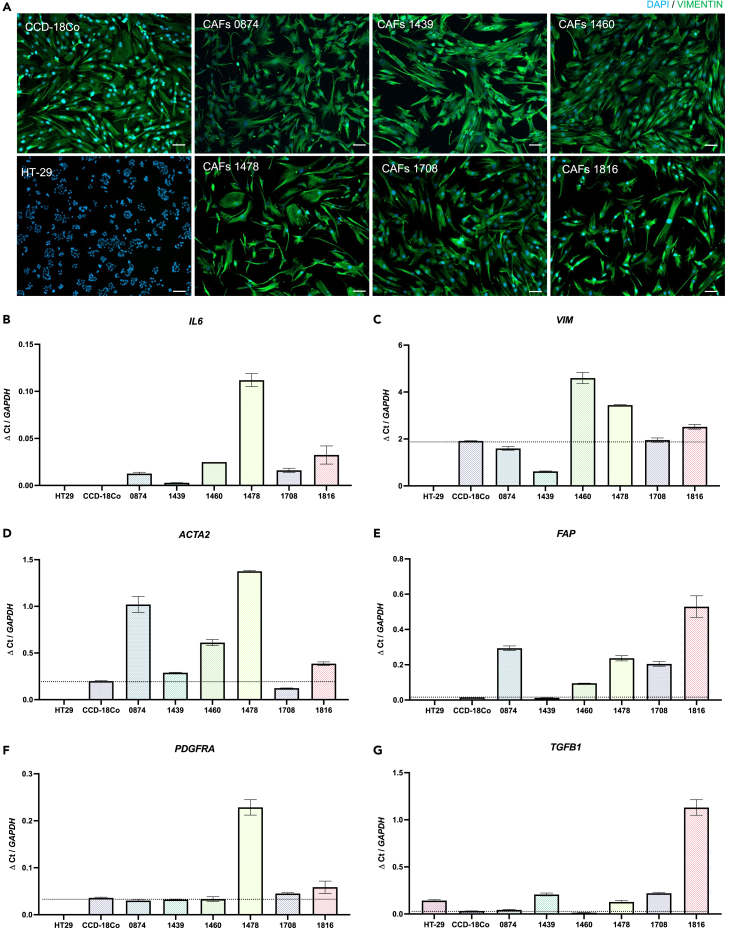
Patient-specific colorectal cancer CAF populations characterization (A) Immunofluorescence staining of 2D CAFs, CCD-18Co (healthy colon fibroblasts), and HT-29 (epithelial colorectal cancer cells). Showing vimentin expression in all CAFs and elongated, spindle-like morphology. Green = vimentin, blue = nuclei. Scale bar: 100 μm. (B–G) Relative mRNA levels of CAF markers (normalized to *GAPDH*), for all six CAFs, HT-29, and CCD-18-Co. Dashed line showing mRNA level of normal colon fibroblast CCD-18Co. (B) Interleukin-6, (C) vimentin, (D) alpha smooth muscle actin, (E) fibroblast activation protein α, (F) PDGFRα, (G) transforming growth factor-β. Data are represented as mean ± SEM.

A recent consensus statement written on CAF characterization states that any mesenchymal cell cultured from a tumor that has an elongated morphology and is positive for a mesenchymal marker, such as vimentin, can be considered CAF.^30^ Upregulation of one or more of the following markers is also indicative of CAF activation, according to the literature: αSMA, vimentin, PDGFRα, FAP, interleukin-6 (IL-6), and TGF-β.^29^^,^^30^^,^^43^^,^^44^ All six CAF samples were characterized in 2D, checking for morphology, vimentin expression, and mRNA upregulation of the markers above. Human normal colon fibroblasts CCD-18Co^45^ and human colorectal epithelial cancer HT-29 cells were used as controls. HT-29^46^ cells should be negative for all gene markers, vimentin expression, and morphological characteristics, whereas CCD-18Co cells should have an elongated morphology and vimentin expression, but lower mRNA levels of CAF activation markers, as they are healthy fibroblasts. Immunofluorescence staining (Figure 2A) showed expression of vimentin in all six samples, along with the healthy colon fibroblasts (CCD-18Co). HT-29 cells did not express vimentin. An elongated morphology was observed in all six CAF samples, similar to the morphology of the normal colon fibroblasts. Looking into more details, the normal, non-activated, non-contractile fibroblasts displayed a homogeneous flat, spindle-shaped morphology.^47^ Sample 0874, 1478, and 1816 showed more heterogeneity in shape and length, where some cells exhibit a thin, wiry morphology and others are large and stellate shaped. This heterogeneity in morphology within one sample may indicate that the CAFs originate from various stromal cell types that have then been activated in cancer-associated fibroblasts.^48^ It may also reflect the different activation stage of individual cells as resting fibroblasts have been reported to be spindle shaped, whereas activated fibroblasts to be generally more stellate shaped, with more lamellipodia protrusion.^49^ Aging, mature CAFs are said to have a thin, way morphology.^49^ Looking at mRNA expression of CAFs markers (Figures 2B–2G), all six samples were positive for *IL-6*, *ACTA2*, *PDGFRA*, *VIM*, *FAP*, and *TGFB1*. HT-29, which are epithelial cancer cells, express none of these markers except *TGFB1*, which is expressed by most cell types under homeostasis and upregulated in cancer cells.^50^ Compared to the expression in normal colon fibroblasts (CCD18-Co), all samples upregulate three or more of these markers. Again, the difference of mRNA expression of these markers between CAF samples speaks to the heterogeneity of CAFs. High *IL-6* expression for example suggests an immunomodulatory role or an immune cell origin (sample 1478).^30^ TGF-β1 was also significantly upregulated compared to the healthy fibroblast (CCD-18Co) at the protein level when the CAF populations were cultured in 3D over 14 days (Figure S1; all *p* > 0.05).

### Heterogeneity in patient-specific CAF remodeling patterns

**Figure 3:**
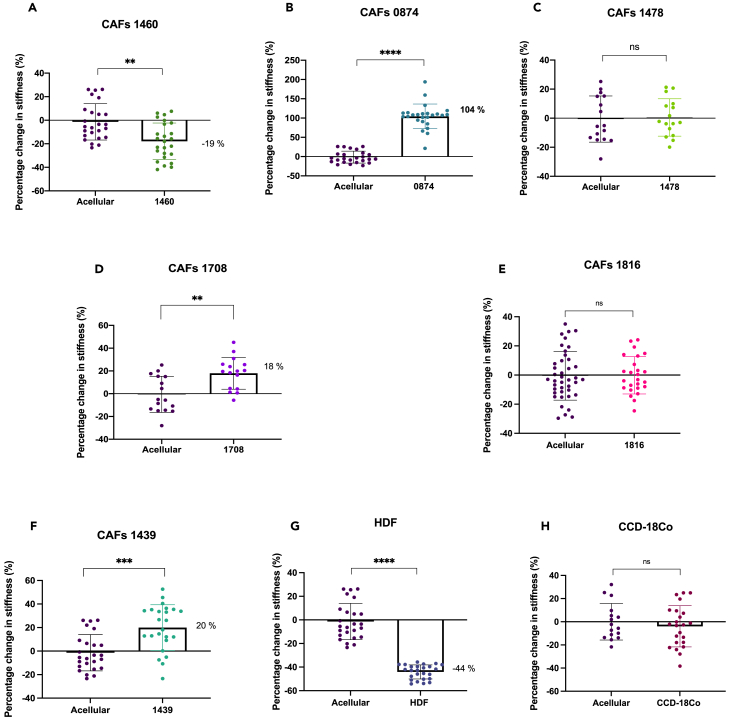
Effect of patient-specific colon CAFs on matrix stiffness over 21 days (A–F) Percentage change in stiffness CAF constructs. (G–H) Percentage change in stiffness of control healthy fibroblast constructs. All measured by AFM (*n* = 3). Percentage change calculated from acellular controls. All *p*-value significance is indicated as: ∗<0.05, ∗∗<0.01, ∗∗∗<0.001, ∗∗∗∗<0.0001. Data are represented as mean ± SD.

Each CAF population was integrated in our biomimetic 3D *in vitro* model, made of a dense collagen matrix, and cultured for 21 days (Figure 1B). On day 21, atomic force microscopy (AFM) measurements were performed to determine the local Young’s modulus and therefore the changes in matrix stiffness caused by each CAF population (Figure 1C). Each construct was set as an *n* = 3 biological replicates, alongside *n* = 3 acellular controls from which to normalize the stiffness measurements. Sixteen measurements per sample were taken along a square grid of 1500 μm^2^. An example of an acquired force curve, from which the apparent Young’s modulus is extracted, can be found in the supplementary section (Figure S2). Significant heterogeneity in remodeling capacities between CAF populations was observed (Figure 3).

Three samples (0874, 1439, and 1708) significantly stiffened their micro-environment (104% stiffening for 0874, 20% stiffening for 1439, and 18% stiffening for 1708, all compared to their acellular controls; *p* < 0.0001, *p* = 0.0004, and *p* = 0.0017, respectively; Figures 3A–3C). CAF samples 1478 and 1816 presented no changes in matrix stiffness compared to the acellular control (Figures 3D and 3E), indicating either no remodeling at all or an equal amount of degradation and matrix deposition/contraction that balanced each other out. This will be in future explored in section 2.3. CAFs 1460, along with control fibroblasts human dermal fibroblasts (HDFs) significantly degraded the matrix (−19% degradation for 1460, −44% degradation for HDFs, both compared to their acellular controls; *p* = 0.0004 and *p* < 0.0001, respectively; Figures 3F and 3G). The healthy colon fibroblasts CCD18-Co showed no changes in stiffness (Figure 3H).

A note to mention that measurement of proliferation rates by metabolic assay showed that none of the CAF samples were highly proliferative in 3D culture, as is expected from primary differentiated mesenchymal cells (Figure S3). This suggests that proliferation rate does not impact the stiffness measurements.

### Both active cell-generated contraction forces and permanent matrix remodeling contribute to the changes in stiffness

**Figure 4:**
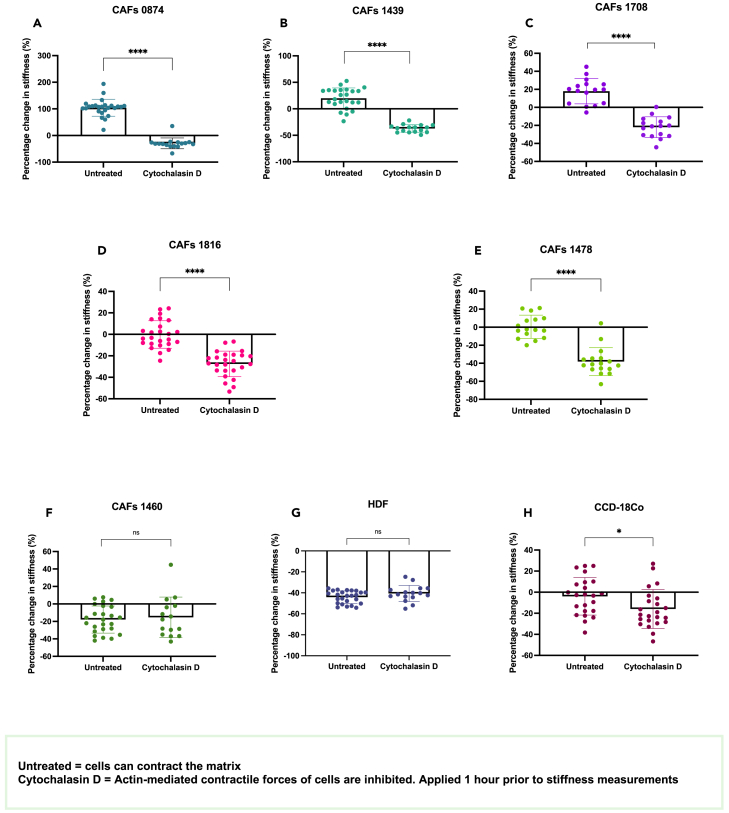
CAF-generated contractile forces contribute to ECM stiffening Percentage change in stiffness prior to (“untreated”) and after 1-h cytochalasin-D treatment of (A–F) CAF constructs. (G–H) Percentage change in stiffness of control healthy fibroblast constructs prior to and after cytochalasin-D treatment. Cytochalasin-D is an actin filaments disrupter. All measured by AFM (*n* = 3). Percentage change normalized to acellular controls. All *p*-value significance is indicated as: ∗<0.05, ∗∗<0.01, ∗∗∗<0.001, and ∗∗∗∗<0.0001. Data are represented as mean ± SD.

Tissue stiffness is dictated not only by ECM protein deposition, cross-linking, and degradation but also by realignment of the fibers and cell-generated tension.^7^^,^^15^^,^^16^^,^^17^^,^^18^^,^^51^ Fibroblast cells in particular are known to generate high contractile forces, which allows them to not only pull and mechanically re-arrange the ECM fibers but also pull on the fibers to migrate.^52^^,^^53^ This tension stiffening of the matrix has been measured in Figure 4. Day 21 constructs were indented by AFM before and after 1 h cytochalasin D treatment, an inhibitor of actin-generated forces.^51^ It was observed that for the CAFs 0874, 1439, 1478, 1708, and 1816 there was a significant decrease in stiffness when the cells could not contract the matrix (*p* < 0.0001 for all three; Figures 4A–4C), indicating that the initial stiffening was due to the contraction of the matrix. CAFs 1460 and healthy HDFs did not generate contractile forces, as shown by the lack of change in stiffness before and after force inhibition (Figures 4F–4G). The healthy CCD-18Co did generate a small but significant amount of contraction (*p* = 0.0293; Figure 4H). Changes in cell compliance induced by cytochalasin D treatment were not thought to have an impact on these results, given our previously published data regarding the impact of cell stiffness on overall matrix stiffness in our 3D models.^2^

**Figure 5:**
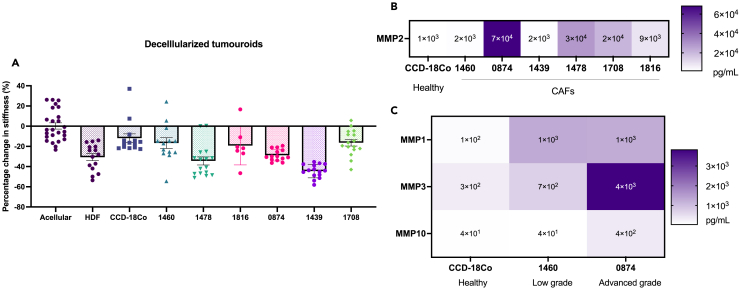
Enzymatic matrix degradation in patient-specific CAF 3D models (A) Percentage change in stiffness after decellularization of day 21 CAF and healthy fibroblast constructs. Normalized to acellular controls. All measured by AFM (*n* = 3). Data are represented as mean ± SD. (B and C) MMPs protein concentration in media collected from constructs at day 14 MMP2 in (B) and MMP1 and 3 and 10 in (C). Average of *n* = 3 biological replicates.

Cells were removed from the matrix, and AFM was performed. The decellularization allows the measure of the ECM stiffness only, without any cells. It informs on the permanent changes in ECM architecture caused by CAFs remodeling. The stiffness of the decellularized constructs was compared to acellular controls, to see how much the CAFs would have degraded or stiffened the matrix over 21 days of culture. It was observed that all the fibroblasts’ (CAFs and healthy controls) decellularized matrices were softer than the acellular gel, meaning that the ECM was degraded (Figure 5A). All the fibroblasts (CAFs and healthy controls) expressed MMP-2 at the protein level after 14 days of culture in the dense collagen matrix, which further implies matrix degradation. The CAFs all secreted more MMP-2 than the healthy colon fibroblast (Figure 5B). Other MMPs such as MMP-1, MMP-3, and MMP-10 were also expressed (Figure 5C) and therefore involved in the degradation of the matrix. MMP-8, MMP-9, MMP-12, and MMP-13 were not expressed in any of the cells (Figure S4).

Together, these data suggest that matrix remodeling by cancer-associated fibroblasts is multi-facetted, with multiple factors operating all at once. Although some of the CAFs stiffen their matrix, thanks to cell-generated matrix contraction, when we look at the matrix itself, without cells, all matrices are softened. This means that both matrix softening and stiffening actions are present at the same time, and the balance of each is unique to each CAF population, leading to different overall tissue stiffness outcomes, as measured in Figure 5. The CAFs likely also deposited new collagen at the same time, allowing for further remodeling of the ECM architecture. As an example of this balance between softening and stiffening factors, CCD-18Co cells degraded the matrix very little, generated little contractile forces (10%, *p* = 0.0293), and the overall stiffness of the matrix after 21 days was not significantly changed compared to the acellular controls. CAFs 1478, however, did overexpress MMPs, and the decellularized constructs were −34% softer than acellular constructs, but they also generated significant tension forces (*p* < 0.0001). The overall stiffness measured in Figure 4 showed no significant changes to matrix stiffness compared to acellular, which can be hypothesized to being due to a perfect balance of matrix stiffening and softening.

### Heterogeneity in remodeling patterns is lost when cocultured with cancer cells, highlighting the impact of the cancer stroma cross-talk

Each primary patient-specific CAF population was cocultured in a dense collagen matrix constructs first with a less invasive colorectal cancer cell line HT-29 and then a more invasive colorectal cancer HCT 116 cell-line and cultured for 21 days. Cancer cell lines were used, allowing to focus on the effect of heterogeneous CAFs on well-characterized, stable, immortalized cancer cells.

**Figure 6:**
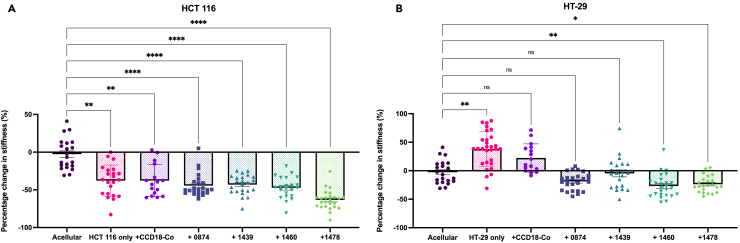
Effect of patient-specific colon CAFs and colorectal cancer cell lines coculture on matrix stiffness over 21 days Percentage change in stiffness of primary patient-specific CAFs and CCD-18Co healthy fibroblasts cultured with either (A) HCT 116 invasive or (B) HT-29 less invasive colorectal cancer cell lines. All measured by AFM (*n* = 3). Percentage change calculated from acellular controls. All *p*-value significance is indicated as: ∗<0.05, ∗∗<0.01, ∗∗∗<0.001, ∗∗∗∗<0.0001. Data are represented as mean ± SD.

On day 21, AFM measurements were performed to determine the changes in stiffness (Figure 6). Three acellular controls, as well as three HCT 116 and three HT-29 constructs in mono-cultures were set up along with the cocultures. As per previously published findings,^2^ the less invasive cells (HT-29) alone significantly stiffened their micro-environment (38% stiffer than the acellular controls, *p* = 0.0044), and the more invasive HCT 116 significantly degraded their matrix (−36% softer than the acellular controls, *p* = 0.0010). It has previously been shown that the observed degradation was linked to an aggressive and invasive phenotype as the cells need to locally degrade the dense matrix to be able to invade out.^2^ The healthy CCD18-Co cocultures exhibited additive stiffness changes. CCD-18 Co alone did not change the overall stiffness, and the HCT 116 degraded the matrix. Together, they also degraded the matrix (−37%). The HT-29 alone stiffened the matrix, and when cultured with CCD18-Co, the matrix was stiffened (+22%).

The CAFs cocultures showed a complete loss of the previously observed heterogeneity in remodeling patterns. When mixed with cancer cells, the measured change in stiffness was degradation for all conditions. The stiffness changes in the matrix were no longer additive, as they were for the healthy fibroblasts. For example, it was established that HT-29 alone stiffened the matrix (by 22%), as did CAFs 0874 (by 104%), but cultured together, these cells did not stiffen the matrix but conversely show a trend of matrix degradation. HCT 116 and CAFs 0874 coculture also degraded the matrix (−44%, *p* < 0.0001), whereas, as mentioned earlier, the CAFs alone stiffened the matrix by 104%. This highlights the impact of cancer cell–stromal cell cross-talk, as cells behaved drastically differently together as opposed to alone. The consistent degradation across all samples matches with the idea that aggressive cancer cells need to locally create holes and paths in the matrix to invade out into the dense collagen matrix. HT-29 cells alone do not degrade the matrix and do not invade much.

## Discussion

Changes in tissue stiffness is one of the hallmarks of cancer.^1^^,^^2^^,^^3^^,^^4^^,^^5^^,^^6^^,^^7^ CAFs are major drivers of the tumor micro-environment (TME) physical remodeling.

To the best of our knowledge, this study is the first to have mechanically characterized the changes in TME stiffness caused by patient-specific CAFs, as well as by CAFs cultured with colorectal cancer cells. To do so we used an engineered, highly biomimetic system, made of dense collagen. The dense matrix correctly replicates the tumor’s native biophysical environment and particularly its stiffness (∼3 kPa).^2^

For this work, CAF samples were successfully isolated and expanded from six different colon tumor biopsies. A 2D culture, with a specialized fibroblast media, was effective at selecting against the growth of other cell populations present in the biopsy (such as epithelial cancer cells, fat or immune cells). Each CAF sample was then thoroughly characterized by confirming morphology, vimentin expression, and mRNA upregulation of six different CAF markers Figure 2).

Each population was then integrated in a construct and cultured for 21 days. Atomic force microscopy (AFM) was used to precisely measure changes in matrix stiffness. AFM measures stiffness (Young’s modulus, E) of a material at the micro-scale by indenting its surface.^46^^,^^47^^,^^48^^,^^49^ This allows for systematic, controlled, high-resolution measures of stiffness, as opposed to other techniques such as rheology or tensile pulling, where only one reading per sample is available, which would be the average stiffness for the whole sample.

This work differs from previously published studies such as by Delaine-Smith et al.^54^ and Jaeschke et al.,^55^ due to their use of immortalized cells (which lack the heterogeneity and complexity of primary cells); less precise stiffness measurement techniques such as bulk rheology, bulk compression, contraction assays, or fiber imaging; and finally due to using a matrix that is not biomimetic in terms of initial ECM stiffness.

The six different patient-derived CAF populations showed heterogeneity in matrix remodeling patterns. Some CAF samples significantly stiffened their matrix, whereas others either did not change matrix stiffness or significantly degraded their micro-environment (Figure 3). The heterogeneity observed herein may initially seem to go against the general consensus that CAFs stiffen the TME.^26^^,^^29^^,^^30^ However, many recent studies highlight the genotypic and phenotypic heterogeneity of CAFs, which dismantles the simplistic view that all CAFs exhibit the same characteristics.^30^^,^^32^^,^^56^ This heterogeneity has herein been shown to also extend to bio-mechanical behaviors.

The stiffening observed is largely due to the contractile forces generated by the CAFs. The observed softening of the matrix is linked to high activity of degradation enzymes such as MMPs. Both contractile forces and MMPs activity has also previously been shown to be heavily involved in matrix remodeling of the TME by cancer cells only.^2^ It is important however to emphasize that one cell population does not display a single “behavior” in terms of only stiffening or only degrading the matrix. This work demonstrates that most of the CAFs both stiffen and soften the matrix. The balance of stiffening actions versus softening actions is what determines the measured overall stiffness of the TME. For example, the intermediate grade CAF 1478 showed overall no changes in matrix stiffness. However, the data here demonstrate that they strongly contracted the matrix (a stiffening action, *p* < 0.0001) but also secreted high amounts of MMPs (softening actions). Both of these canceled each other out and resulted in no directly measurable changes in matrix stiffness. Matrix remodeling is a complex process involving many simultaneous actions such as fiber deposition, cross-linking, re-alignment, cell-generated tension forces, matrix degradation etc., and the work here shows that not all CAFs remodel their ECM the same way. Future work will include measuring fiber deposition for each CAF population, as well as visualizing collagen alignment.

This heterogeneity was lost when the CAFs were cocultured with colorectal cancer cell lines. All six CAFs samples, with either HT-29 or HCT 116 cells, significantly degraded their micro-environment and softened the 3D matrix. This degradation is linked to an enhanced epithelial to mesenchymal transition (EMT) in the cancer cells.^2^

This is believed to be caused by invasive cancer cells needing to locally create holes and paths in the matrix to invade out into the dense collagen environment.^57^^,^^58^ The degradation patterns observed above using CAF-cancer cells 3D models correlate with observations made on biopsy samples.^14^^,^^59^ This highlights the impact of the cross-talk between cancer cells and CAFs, and the importance of integrating both cell population in any colorectal cancer model.

### Limitations of the study

The main limitation of this study is the restricted amount of CAF populations. Using primary, patient-derived cells provide unique insights into CAF behavior as they better represent the heterogeneity of human cancer biology. However, acquiring tissues from consenting patients and then successfully isolating and expanding the cells is a difficult and tedious process. Furthermore, we do not get to choose the biopsies’ cancer grade, as the pathological report is not yet available at the time of the samples’ collection. We therefore present this work with a total of six unique CAF populations, each isolated from a different patient.

A further limitation of using primary, nonimmortalized cells is that, were we to repeat the experiments, a slight variation in results may occur for each CAF sample. Indeed, non-immortalized cells differentiate, and therefore, a repeat will have cells differentiating differently/at different paces. This is mitigated by the use of cells up to passage five only but given that we culture the constructs for 21 days, differentiation is bound to occur. The results presented earlier are therefore not intended for strong conclusion of the remodeling ability of one patient’s CAF but more so to highlight the heterogeneity from person to person and tumor to tumor.

## STAR★Methods

### Key resources table

**Table:** 

REAGENT or RESOURCE	SOURCE	IDENTIFIER
**Antibodies**		

Anti-Vimentin mouse V9	Santa Cruz, Texas, US	Santa Cruz Biotechnology Cat# sc-6260 ; RRID: AB_628437
Anti-mouse Alexa Fluor™ 488 IgG H&L	Abcam, Cambridge, UK)	Abcam Cat# ab150113 ; RRID: AB_2576208

**Chemicals, peptides, and recombinant proteins**		

L-ascorbic acid 2-phosphate	Sigma Aldrich, Dorset, UK	Cat# A8960
Rat-tail collagen type I (2.05 mg/ml in 0.6% acetic acid)	First Link, Birmingham, UK	Cat# 60-30-810
Epidermal growth factor (EGF), PeproTech	Gibco, Thermo Fisher Scientific, Loughborough, UK	Cat# AF-100-15-1MG
TheraPEAK™ ACK Lysing Buffer	Lonza, Slough, UK	Cat# BP10-548E
TRI Reagent™ Solution	Thermo Fisher Scientific, Loughborough, UK	Cat# AM9738
Cytochalasin D	Thermo Fisher Scientific, Loughborough, UK	Cat# PHZ1063

**Critical commercial assays**		

Tumour dissociation kit, human	Miltenyi Biotec, Cologne, Germany	Cat# 130-095-929
RAFT™ absorbers	Lonza, Slough, UK	Cat# 016-1R16
High-Capacity cDNA Reverse Transcriptase Kit	Thermo Fisher Scientific, Loughborough, UK	Cat# 368814
iTaq Universal SYBR Green Super mix	Bio-Rad, Hertfordshire, UK	Cat# 1725124
LIVE/DEAD reagents	Thermo Fisher Scientific, Loughborough, UK	Cat# L-3224
NucBlue™ Fixed Cell ReadyProbes™ Reagent (DAPI)	Thermo Fisher Scientific, Loughborough, UK	Cat# R37606

**Experimental models: Cell lines**		

HT-29	ECACC, Salisbury, United Kingdom	Cat# 91072201
HCT116	ECACC, Salisbury, United Kingdom	Cat# 91091005
CCD-18Co	ATCC, MNZ, VA, USA	Cat# CRL-1459
HDF	Thermo Fisher Scientific, Loughborough, UK	Cat# C0135C

**Oligonucleotides**		

Primers for *IL6* Forward: TTGCTGTTATTGTGGTTGTGGTG	BJC Pape 2020[56]	N/A
Primers for *IL6* Reverse: CCCATCTCCTTTATCTCAGCCTTC	BJC Pape 2020[56]	N/A
Primers for *VIM* Forward: TCTCTGGCACGTCTTGACCTTG	AHM Micalet 2022[2]	N/A
Primers for *VIM* Reverse: CGATTTGGACATGCTGTTCCTG	AHM Micalet 2022[2]	N/A
Primers for *ACTA2* Forward: CAGGAGGAGAAGGCTGTGTTC	BJC Pape 2020[56]	N/A
Primers for *ACTA2* Reverse: TAAAGGCGGCATCCACTCG	BJC Pape 2020[56]	N/A
Primers for *FAP* Forward: CAGTCCACCCTTGTGCTCTTCC	BJC Pape 2020[56]	N/A
Primers for *FAP* Reverse: TTCGACTCTCCACGCATCTCTG	BJC Pape 2020[56]	N/A
Primers for *PDGFRA* Forward: TACTTCCACAGGTCCCACAACC	BJC Pape 2020[56]	N/A
Primers for *PDGFRA* Reverse: GCATTCCTCACAGCCAACAGTG	BJC Pape 2020[56]	N/A
Primers for *TGFB1* Forward: CACCAACTATTGCTTCAGCTCCAC	This paper	N/A
Primers for *TGFB1* Reverse: TGTCCAGGCAAATGTAGGG	This paper	N/A
Primers for *GAPDH* Forward: GCTCTCTGCTCCTCCTGTTC	Al Hosni iScience 2022[57]	N/A
Primers for *GAPDH* Reverse: CGACCAAATCCGTTGACTCC	Al Hosni iScience 2022[57]	N/A

**Software and algorithms**		

ImageJ	ImageJ	National Institute of Health, RRID:SCR_003070 (https://imagej.nih.gov/ij/)
GraphPad Prism version 10	GraphPad Prism Software, La Jolla, CA, USA	https://graphpad.com
JPK BioAFM SPM data processing software	Bruker, Berlin, Germany	N/A

**Other**		

AFM cantilevers RFESP-75	Bruker, Berlin, Germany	Cat# RFESP-75

### Resource availability

#### Lead contact

Further information and requests for resources and reagents should be directed to and will be fulfilled by the lead contact, Umber Cheema (u.cheema@ucl.ac.uk).

#### Materials availability

This study did not generate new unique reagents.

#### Data and code availability

• All data reported in this paper will be shared by the lead contact upon request.
• This paper does not report original code.
• Any additional information required to reanalyse the data reported in this paper is available from the lead contact upon request.

### Experimental model and study participant details

#### Isolation of primary cancer associated fibroblasts (CAFs)

Colorectal tumour biopsies (∼1g) were collected, with informed consent, from patients at the Royal Free Hospital, London, UK (ethics number 21/WA/0388). Patient information, including gender, can be found in supplementary Table S1. Fresh tissue was first washed in phosphate-buffered saline (PBS) with 1% antibiotic-antimycotic. The tissue was then cut up in small pieces in a petri dish. Wash media (DMEM high glucose, 1% AA) was added to the petri dish, along with enzymes from the tumour dissociation kit (human, Miltenyi Biotec, Cologne, Germany). The petri dish was placed on a shaker, in an incubator, for an hour. After, the cell/media/tissue mix was strained, and the final solution centrifuged. The cell pellet was resuspended in red blood cell (RBC) lysis buffer (Lonza, Slough, UK). The solution was placed in the water bath for 2 minutes, then fresh media was added to the tube to neutralise the buffer and the tube was centrifuged again. The supernatant was once again removed and the cells resuspended in culture media (DMEM high glucose, 1% AA, 20% FBS, 20 ng/mL epidermal growth factor (EGF)^60^^,^^61^), and placed in a flask for culture. The 2D conditions, along with this specific media, ensure that only fibroblasts survive and proliferated. All media and media supplements were from Gibco™, Thermo Fisher Scientific, Loughborough, UK.

#### Cell culture

Human colorectal cancer cell lines HT-29 and HCT 116 were obtained from the ECACC (ECACC Cat# 91072201 and 91091005 respectively, through Sigma Aldrich, Dorset, UK). The human normal colon fibroblast cell line CCD-18Co were purchased from the ATCC (ATCC Cat# CRL-1459, through LGC standards, Middlesex, UK). The human dermal fibroblast HDF were purchased from Invitrogen™ (Cat# C0135C, through Thermo Fisher Scientific, Loughborough). The primary, patient-specific CAFs were isolated as per the section above. HT-29 and HCT 116 cells were cultured in low glucose DMEM (1g/L) supplemented with 10% foetal bovine serum (FBS) and 1% PenStrep. CCD-18Co and HDF cells were cultured in high glucose DMEM (5 g/L) supplemented with 10% FBS and 1% PenStrep. The CAFs were cultured in high glucose DMEM supplemented with 20% FBS, 1% antibiotic-antimycotic, and 20 ng/mL EGF. All reagents were provided from Gibco™ through Thermo Fisher Scientific, Loughborough, UK. Cells were cultured at 5% carbon dioxide (CO_2_) atmospheric pressure and at 37^o^C and passaged regularly in 2D monolayers. CAFs and HDF were used up to passage 5 to avoid differentiation. Immortalized cell lines HT-29, HCT 116 were used between passage 10 and 20; CCD-18Co were used between passage 0 and 10. All cells were routinely tested for mycoplasma.

#### Engineering of simple 3D compressed collagen constructs

Constructs (3D models) made of a dense collagen matrix were engineered according to a method previously described.^2^ Briefly, 2 mg/mL collagen type I hydrogels were set up using monomeric rat-tail collagen type-1 (First Link, Birmingham, UK), 10X MEM (Sigma Aldrich, Dorset, UK), and a neutralizing solution. Cells were added to the collagen mix at concentrations found in Table S2. The cell ratio used has been previously established.^42^ To set the hydrogels, 1.3 mL of the collagen mix was pipetted in a 24 well-plate well and then polymerised for 15 min at 37^o^C. The hydrogels were then compressed for 15 min using RAFT™ absorbers (Lonza, Slough, UK). RAFT™ absorbers cause permanent removal of the fluid to create stable, dense collagen constructs.^35^ The compression does not affect cell viability.^36^^,^^62^ The constructs were cultured for 21 days at 5% CO_2_, 37^o^C. The media was supplemented with ascorbic acid 50 μg/ml (Sigma-Aldrich, Dorset, UK). 50% media changes were performed every 48 hours to allow growth factors released by the cells to remain present. The reproducibility of the 3D constructs is shown supplementary Figure S5.

#### Ethics statement

All methods were carried out in accordance with relevant guidelines and regulations. Patient samples were obtained, with informed consent, from patients with colorectal tumours through the Tissue Access for Patient Benefit initiative (TAPb) at The Royal Free Hospital, London, UK. The ethics was approved by the University College London Royal Free Hospital BioBank Ethical Review Committee; Research Ethics Committee Reference number 21/WA/0388).

### Method details

#### RNA extraction and quantitative polymerase chain reaction (qPCR)

RNA was extracted from CAFs, HT-29 and CCD-18Co cells grown in 2D in a T75 flask. Phase separation TRI Reagent® and chloroform method was used.^63^ cDNA was transcribed using the High-Capacity cDNA Reverse Transcription Kit (Applied Biosystems™ through, Fisher Scientific, Loughborough, UK). Primers used for qPCR have been previously published, and can be found listed in Table S3. qPCR was performed using iTaq™ Universal SYBR® Green Supermix (Applied Biosystems™ through, Fisher Scientific, Loughborough, UK). Three technical replicates were performed. Relative gene expression was calculated using the ΔCt method, normalising to house-keeping gene GAPDH.^64^

#### Cytokine analysis

Media was collected at day 14 from the constructs (n=3 biological replicates). The media was sent to Eve Technologies (Calgary, Canada) where luminex assays were performed to determine protein concentration of TGFβ-1 and a range of MMPs.

#### Immunofluorescent staining and imaging

For CAFs characterisation, cells were seeded at 500,000 cells/well in a 6 well plate (n=3). After 24h, the cells were fixed using 10% neutrally buffered formalin (Genta Medical, York, UK) for 30 min and then washed and stored in PBS (Gibco™, through Fisher Scientific, Loughborough, UK). Before staining, all samples were blocked for 1h using 0.2% triton X-100 and 1% bovine serum albumin (BSA) (both from Sigma-Aldrich, Dorset, UK) in PBS. Then, the primary antibody, diluted in blocking solution (see Table S4 for dilutions), was applied to the samples. They were incubated overnight at 4^o^C. The secondary antibody incubation was carried out the next day for 2.5 hours, at room temperature. A DAPI counterstain was applied 20 min before imaging (NucBlue™, Invitrogen™ through Fisher Scientific, Loughborough, UK). Samples were imaged on the Zeiss AxioObserver and Zeiss ZEN software (Zeiss, Oberkochen, Germany).

#### Atomic force microscopy

AFM was performed on simple constructs (n=3), after 21 days of culture. Each experiment also had n=3 acellular controls. The samples were left in a humidified incubator (37^o^C) right until measurements were taken. Cell culture media was changed to Leibovitz's L-15, no phenol red, imaging media (Gibco™ through Thermo Fisher Scientific, Loughborough, UK). Measurements were performed in liquid, at room temperature. A CellHesion® 200 AFM (JPK BioAFM, Bruker Nano GmbH, Berlin, Germany) was used. The cantilever used was a RFESP-75 (k ∼ 2 N/m, Bruker, Berlin, Germany) with a 50 μm of diameter glued glass bead (Cospheric LLC, California, USA). It was calibrated in liquid, on glass, on the day of the experiment, to determine sum and sensitivity. Each construct was probed along a grid (4 x 4 map of 1500 x 1500 μm leading to a total of 16 measurements per sample). The set force was 700 nN, which insures a 10 to 15 μm indentation, which is less than 10% of the thickness of our samples (150-200 μm).^65^ Due to variabilities introduced by AFM calibration, for each experiment n=3 acellular control gels were measured by AFM, to which we then normalised the measured AFM data of cellular constructs. Using the JPK BioAFM SPM data processing software, the Hertz model was fitted to the collected force curves to determine the apparent Young’s Modulus E, assuming a Poisson ratio (ν) of 0.5.^66^

#### Cytochalasin D treatment and decellularization

Cytochalasin D, an actin filaments disrupter (Invitrogen™, through Thermo Fisher Scientific, Loughborough) was used at 20 μM diluted in media with 0.1% DMSO vehicle control and applied for 1 h, to both cellular and acellular samples (controls). Cytochalasin D was present during the AFM measurements, as its effect is reversible. Decellularization was achieved using 0.5% triton X-100 (Sigma-Aldrich, Dorset, UK) and 11 mM ammonium hydroxide (Sigma-Aldrich, Dorset, UK) in PBS for 1 h under gentle agitation.

### Quantification and statistical analysis

All data was analysed and visualised using GraphPad Prism 9 software. All AFM data was non-parametric, therefore Mann-Whitney (2 groups) or Kruskal-Wallis (three groups or more) tests were used. All n numbers, p-values and tests conducted are mentioned in figure caption. N numbers represents number of experimental replicates for each condition. P-value significance is indicated as: 0.05 < ∗, 0.01 <∗∗, 0.001 < ∗∗∗ and 0.0001 < ∗∗∗∗.

## References

[1] Micalet A., Moeendarbary E., Cheema U. (2023). 3D *In Vitro* Models for Investigating the Role of Stiffness in Cancer Invasion. ACS Biomater. Sci. Eng..

[2] Micalet A., Pape J., Bakkalci D., Javanmardi Y., Hall C., Cheema U., Moeendarbary E. (2023). Evaluating the Impact of a Biomimetic Mechanical Environment on Cancer Invasion and Matrix Remodeling. Adv. Healthc. Mater..

[3] Hanahan D., Weinberg R.A. (2011). Hallmarks of cancer: The next generation. Cell.

[4] Pape J., Micalet A., Alsheikh W., Ezbakh N., Virjee R.I., Al Hosni R., Moeendarbary E., Cheema U. (2023). Biophysical Parameters Can Induce Epithelial-to-Mesenchymal Phenotypic and Genotypic Changes in HT-29 Cells: A Preliminary Study. Int. J. Mol. Sci..

[5] Nagelkerke A., Bussink J., Rowan A.E., Span P.N. (2015). The mechanical microenvironment in cancer: How physics affects tumours. Semin. Cancer Biol..

[6] Almagro J., Messal H.A., Elosegui-Artola A., van Rheenen J., Behrens A. (2022). Tissue architecture in tumor initiation and progression. Trends Cancer.

[7] Butcher D.T., Alliston T., Weaver V.M. (2009). A tense situation: Forcing tumour progression. Nat. Rev. Cancer.

[8] Chen M.B., Javanmardi Y., Shahreza S., Serwinski B., Aref A., Djordjevic B., Moeendarbary E. (2023). Mechanobiology in oncology: basic concepts and clinical prospects. Front. Cell Dev. Biol..

[9] Wen B., Xu L.Y., Li E.M. (2020). LOXL2 in cancer: regulation, downstream effectors and novel roles. Biochim. Biophys. Acta. Rev. Cancer.

[10] Salvador F., Martin A., López-Menéndez C., Moreno-Bueno G., Santos V., Vázquez-Naharro A., Santamaria P.G., Morales S., Dubus P.R., Muinelo-Romay L. (2017). Lysyl oxidase-like protein LOXL2 promotes lung metastasis of breast cancer. Cancer Res..

[11] Peinado H., Del Carmen Iglesias-de la Cruz M., Olmeda D., Csiszar K., Fong K.S.K., Vega S., Nieto M.A., Cano A., Portillo F. (2005). A molecular role for lysyl oxidase-like 2 enzyme in Snail regulation and tumor progression. EMBO J..

[12] Ferreira S., Saraiva N., Rijo P., Fernandes A.S. (2021). Loxl2 inhibitors and breast cancer progression. Antioxidants.

[13] Amendola P.G., Reuten R., Erler J.T. (2019). Interplay between LOX enzymes and integrins in the tumor microenvironment. Cancers.

[14] Conklin M.W., Eickhoff J.C., Riching K.M., Pehlke C.A., Eliceiri K.W., Provenzano P.P., Friedl A., Keely P.J. (2011). Aligned collagen is a prognostic signature for survival in human breast carcinoma. Am. J. Pathol..

[15] Ahmadzadeh H., Webster M.R., Behera R., Jimenez Valencia A.M., Wirtz D., Weeraratna A.T., Shenoy V.B. (2017). Modeling the two-way feedback between contractility and matrix realignment reveals a nonlinear mode of cancer cell invasion. Proc. Natl. Acad. Sci. USA.

[16] Northcott J.M., Dean I.S., Mouw J.K., Weaver V.M. (2018). Feeling stress: The mechanics of cancer progression and aggression. Front. Cell Dev. Biol..

[17] Kraning-Rush C.M., Califano J.P., Reinhart-King C.A. (2012). Cellular Traction Stresses Increase with Increasing Metastatic Potential. PLoS One.

[18] Mohammadi H., Sahai E. (2018). Mechanisms and impact of altered tumour mechanics. Nat. Cell Biol..

[19] Micalet A., Tappouni L.J., Peszko K., Karagianni D., Lam A., Counsell J.R., Quezada S.A., Moeendarbary E., Cheema U. (2023). Urokinase-type plasminogen activator (uPA) regulates invasion and matrix remodelling in colorectal cancer. Matrix Biol. Plus.

[20] Salem N., Kamal I., Al-Maghrabi J., Abuzenadah A., Peer-Zada A.A., Qari Y., Al-Ahwal M., Al-Qahtani M., Buhmeida A. (2016). High expression of matrix metalloproteinases: MMP-2 and MMP-9 predicts poor survival outcome in colorectal carcinoma. Future Oncol..

[21] Zucker S., Vacirca J. (2004). Role of matrix metalloproteinases (MMPs) in colorectal cancer. Cancer Metastasis Rev..

[22] Agrawal A., Shahreza S., Javanmardi Y., Szita N., Moeendarbary E. (2022). The tumour microenvironment modulates cancer cell intravasation. Organs-on-a-Chip.

[23] Wei S.C., Fattet L., Tsai J.H., Guo Y., Pai V.H., Majeski H.E., Chen A.C., Sah R.L., Taylor S.S., Engler A.J., Yang J. (2015). Matrix stiffness drives epithelial-mesenchymal transition and tumour metastasis through a TWIST1-G3BP2 mechanotransduction pathway. Nat. Cell Biol..

[24] Dai J., Qin L., Chen Y., Wang H., Lin G., Li X., Liao H., Fang H. (2019). Matrix stiffness regulates epithelial-mesenchymal transition via cytoskeletal remodeling and MRTF-A translocation in osteosarcoma cells. J. Mech. Behav. Biomed. Mater..

[25] Catalano V., Turdo A., Di Franco S., Dieli F., Todaro M., Stassi G. (2013). Tumor and its microenvironment: A synergistic interplay. Semin. Cancer Biol..

[26] Bussard K.M., Mutkus L., Stumpf K., Gomez-Manzano C., Marini F.C. (2016). Tumor-associated stromal cells as key contributors to the tumor microenvironment. Breast Cancer Res..

[27] Javanmardi Y., Agrawal A., Malandrino A., Lasli S., Chen M., Shahreza S., Serwinski B., Cammoun L., Li R., Jorfi M. (2023). Endothelium and Subendothelial Matrix Mechanics Modulate Cancer Cell Transendothelial Migration. Adv. Sci..

[28] Agrawal A., Lasli S., Javanmardi Y., Coursier D., Micalet A., Watson S., Shahreza S., Serwinski B., Djordjevic B., Szita N. (2023). Stromal cells regulate mechanics of tumour spheroid. Mater. Today Bio.

[29] Asif P.J., Longobardi C., Hahne M., Medema J.P. (2021). The Role of Cancer-Associated Fibroblasts in Cancer Invasion and Metastasis. Cancers.

[30] Sahai E., Astsaturov I., Cukierman E., DeNardo D.G., Egeblad M., Evans R.M., Fearon D., Greten F.R., Hingorani S.R., Hunter T. (2020). A framework for advancing our understanding of cancer-associated fibroblasts. Nat. Rev. Cancer.

[31] Gaggioli C., Hooper S., Hidalgo-Carcedo C., Grosse R., Marshall J.F., Harrington K., Sahai E. (2007). Fibroblast-led collective invasion of carcinoma cells with differing roles for RhoGTPases in leading and following cells. Nat. Cell Biol..

[32] Venning F.A., Zornhagen K.W., Wullkopf L., Sjölund J., Rodriguez-Cupello C., Kjellman P., Morsing M., Hajkarim M.C., Won K.J., Erler J.T., Madsen C.D. (2021). Deciphering the temporal heterogeneity of cancer-associated fibroblast subpopulations in breast cancer. J. Exp. Clin. Cancer Res..

[33] Qi J., Sun H., Zhang Y., Wang Z., Xun Z., Li Z., Ding X., Bao R., Hong L., Jia W. (2022). Single-cell and spatial analysis reveal interaction of FAP+ fibroblasts and SPP1+ macrophages in colorectal cancer. Nat. Commun..

[34] Luo H., Xia X., Huang L.B., An H., Cao M., Kim G.D., Chen H.N., Zhang W.H., Shu Y., Kong X. (2022). Pan-cancer single-cell analysis reveals the heterogeneity and plasticity of cancer-associated fibroblasts in the tumor microenvironment. Nat. Commun..

[35] Brown R.A., Wiseman M., Chuo C.B., Cheema U., Nazhat S.N. (2005). Ultrarapid engineering of biomimetic materials and tissues: Fabrication of nano- and microstructures by plastic compression. Adv. Funct. Mater..

[36] Cheema U., Brown R.A. (2013). Rapid Fabrication of Living Tissue Models by Collagen Plastic Compression: Understanding Three-Dimensional Cell Matrix Repair *In Vitro*. Adv. Wound Care.

[37] Pape J., Magdeldin T., Ali M., Walsh C., Lythgoe M., Emberton M., Cheema U. (2019). Cancer invasion regulates vascular complexity in a three-dimensional biomimetic model. Eur. J. Cancer.

[38] Pape J., Stamati K., Al Hosni R., Uchegbu I.F., Schatzlein A.G., Loizidou M., Emberton M., Cheema U. (2021). Tissue-Engineering the Fibrous Pancreatic Tumour Stroma Capsule in 3D Tumouroids to Demonstrate Paclitaxel Response. Int. J. Mol. Sci..

[39] Bakkalci D., Jay A., Rezaei A., Howard C.A., Haugen H.J., Pape J., Kishida S., Kishida M., Jell G., Arnett T.R. (2021). Bioengineering the ameloblastoma tumour to study its effect on bone nodule formation. Sci. Rep..

[40] Brauchle E., Kasper J., Daum R., Schierbaum N., Falch C., Kirschniak A., Schäffer T.E., Schenke-Layland K. (2018). Biomechanical and biomolecular characterization of extracellular matrix structures in human colon carcinomas. Matrix Biol..

[41] Deptuła P., Łysik D., Pogoda K., Cieśluk M., Namiot A., Mystkowska J., Król G., Głuszek S., Janmey P.A., Bucki R. (2020). Tissue Rheology as a Possible Complementary Procedure to Advance Histological Diagnosis of Colon Cancer. ACS Biomater. Sci. Eng..

[42] Pape J., Magdeldin T., Stamati K., Nyga A., Loizidou M., Emberton M., Cheema U. (2020). Cancer-associated fibroblasts mediate cancer progression and remodel the tumouroid stroma. Br. J. Cancer.

[43] Biffi G., Tuveson D.A. (2021). Diversity and Biology of Cancer-Associated Fibroblasts. Physiol. Rev..

[44] Han C., Liu T., Yin R. (2020). Biomarkers for cancer-associated fibroblasts. Biomark. Res..

[45] CCD-18Co - CRL-1459 | ATCC https://www.atcc.org/products/crl-1459.

[46] HT-29 - HTB-38 | ATCC https://www.atcc.org/products/htb-38.

[47] Liu T., Zhou L., Li D., Andl T., Zhang Y. (2019). Cancer-associated fibroblasts build and secure the tumor microenvironment. Front. Cell Dev. Biol..

[48] Gunaydin G. (2021). CAFs Interacting With TAMs in Tumor Microenvironment to Enhance Tumorigenesis and Immune Evasion. Front. Oncol..

[49] Son G.M., Kwon M.S., Shin D.H., Shin N., Ryu D., Kang C.D. (2019). Comparisons of cancer-associated fibroblasts in the intratumoral stroma and invasive front in colorectal cancer. Medicine.

[50] Hao Y., Baker D., Ten Dijke P. (2019). TGF-β-Mediated Epithelial-Mesenchymal Transition and Cancer Metastasis. Int. J. Mol. Sci..

[51] Whisler J., Shahreza S., Schlegelmilch K., Ege N., Javanmardi Y., Malandrino A., Agrawal A., Fantin A., Serwinski B., Azizgolshani H. (2023). Emergent mechanical control of vascular morphogenesis. Sci. Adv..

[52] Vicente-Manzanares M., Horwitz A.R. (2011). Cell Migration: An Overview. Methods Mol. Biol..

[53] Blanchoin L., Boujemaa-Paterski R., Sykes C., Plastino J. (2014). Actin dynamics, architecture, and mechanics in cell motility. Physiol. Rev..

[54] Delaine-Smith R., Wright N., Hanley C., Hanwell R., Bhome R., Bullock M., Drifka C., Eliceiri K., Thomas G., Knight M. (2019). Transglutaminase-2 Mediates the Biomechanical Properties of the Colorectal Cancer Tissue Microenvironment that Contribute to Disease Progression. Cancers.

[55] Jaeschke A., Jacobi A., Lawrence M.G., Risbridger G.P., Frydenberg M., Williams E.D., Vela I., Hutmacher D.W., Bray L.J., Taubenberger A. (2020). Cancer-associated fibroblasts of the prostate promote a compliant and more invasive phenotype in benign prostate epithelial cells. Mater. Today Bio.

[56] Yu Z., Zhang J., Zhang Q., Wei S., Shi R., Zhao R., An L., Grose R., Feng D., Wang H. (2022). Single-cell sequencing reveals the heterogeneity and intratumoral crosstalk in human endometrial cancer. Cell Prolif..

[57] Fang M., Yuan J., Peng C., Li Y. (2014). Collagen as a double-edged sword in tumor progression. Tumour Biol..

[58] Winkler J., Abisoye-Ogunniyan A., Metcalf K.J., Werb Z. (2020). Concepts of extracellular matrix remodelling in tumour progression and metastasis. Nat. Commun..

[59] Plodinec M., Loparic M., Monnier C.A., Obermann E.C., Zanetti-Dallenbach R., Oertle P., Hyotyla J.T., Aebi U., Bentires-Alj M., Lim R.Y.H., Schoenenberger C.A. (2012). The nanomechanical signature of breast cancer. Nat. Nanotechnol..

[60] Kim D., Kim S.Y., Mun S.K., Rhee S., Kim B.J. (2015). Epidermal growth factor improves the migration and contractility of aged fibroblasts cultured on 3D collagen matrices. Int. J. Mol. Med..

[61] Yu A., Matsuda Y., Takeda A., Uchinuma E., Kuroyanagi Y. (2012). Effect of EGF and bFGF on fibroblast proliferation and angiogenic cytokine production from cultured dermal substitutes. J. Biomater. Sci. Polym. Ed..

[62] Al Hosni R., Bozec L., Roberts S.J., Cheema U. (2022). Reprogramming bone progenitor identity and potency through control of collagen density and oxygen tension. iScience.

[63] Rio D.C., Ares M., Hannon G.J., Nilsen T.W. (2010). Purification of RNA using TRIzol (TRI reagent). Cold Spring Harb. Protoc..

[64] Schmittgen T.D., Livak K.J. (2008). Analyzing real-time PCR data by the comparative CT method. Nat. Protoc..

[65] JPK Instruments (2014). Determining the elastic modulus of biological samples using atomic force microscopy. https://www.jpk.com/app-technotes-img/AFM/pdf/jpk-app-elastic-modulus.14-1.pdf.

[66] Javanmardi Y., Colin-York H., Szita N., Fritzsche M., Moeendarbary E. (2021). Quantifying cell-generated forces: Poisson’s ratio matters. Commun. Phys..

