# Supplementary material for: Patient-specific colorectal-cancer-associated fibroblasts modulate tumor microenvironment mechanics
Source: iScience. 2024 May 21;27(6):110060. doi: 10.1016/j.isci.2024.110060 (PMC11179580; doi:10.1016/j.isci.2024.110060)
Supplement: Document S1. Figures S1–S5 and Tables S1–S4 [file mmc1.pdf]

iScience, Volume *27*

Supplemental information

Patient-specific colorectal-cancer-associated fibroblasts modulate tumor microenvironment mechanics

Auxtine Micalet, Anuja Upadhyay, Yousef Javanmardi, Camila Gabriela de Brito, Emad Moeendarbary, and Umber Cheema

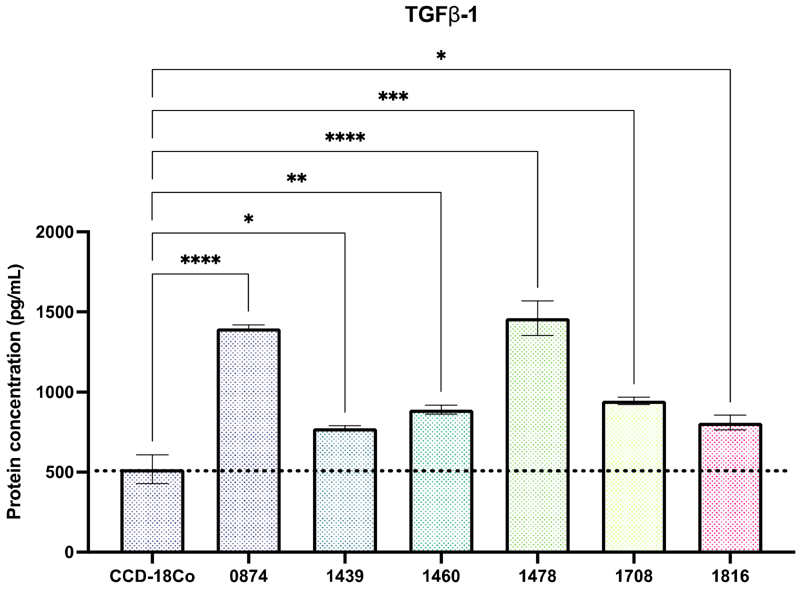

Figure S1 – TGFβ-1 protein expression in each CAF populations, when cultured for 14 days in a dense collagen, related to Figure 2. Dashed line showing protein level of normal colon fibroblast CCD-18Co. n=3 biological replicated. All p-value significance is indicated as: * <0.05, ** <0.01, *** <0.001, **** <0.0001.

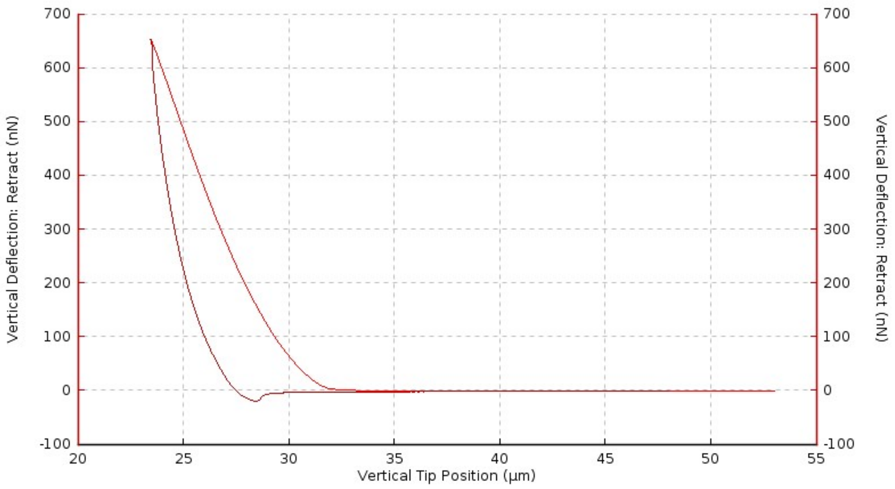

Figure S2 – Representative force-curve obtained by AFM, related to Figure 3. Force-curve obtained by AFM measurements of sample 1708 (biological replicate n1).

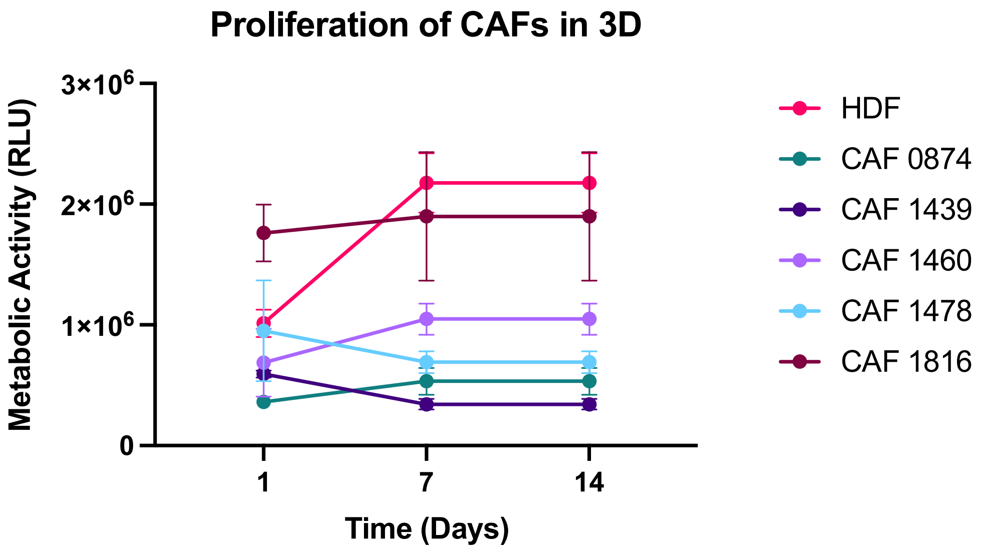

Figure S3 – Proliferation of our primary CAF populations in 3D, related to Figure 3. Measured by CellTiter Glo 3D Cell Viability Assay. n=3 biological replicated. Data are represented as mean +/- SEM.

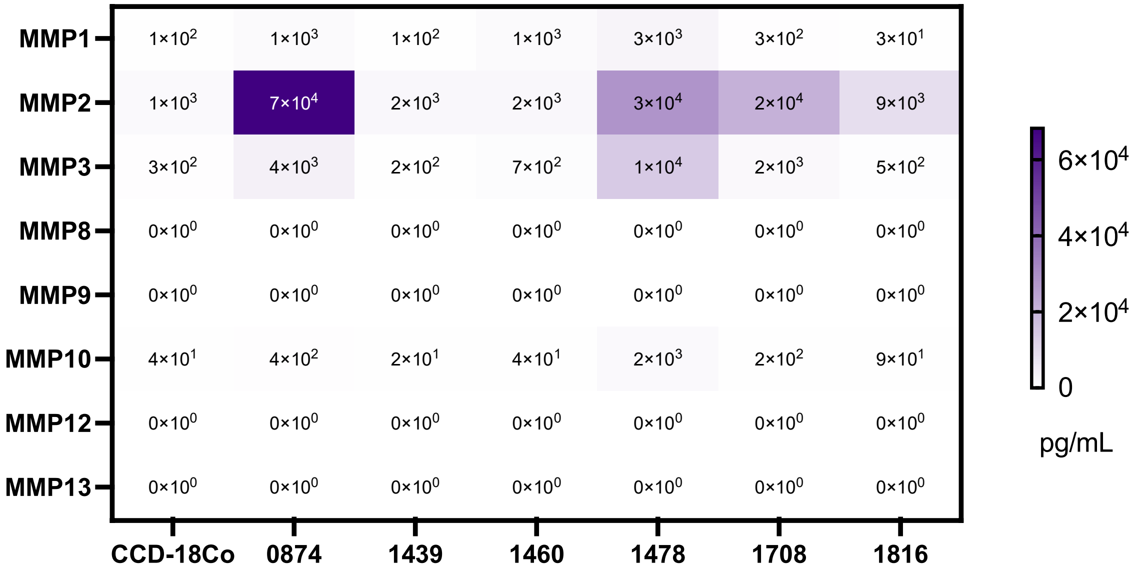

Figure S4 – MMP protein expression in each CAF populations, when cultured for 14 days in dense collagen, related to Figure 5. MMPs protein concentration in media collected from constructs at day 14. Average of n=3 biological replicates.

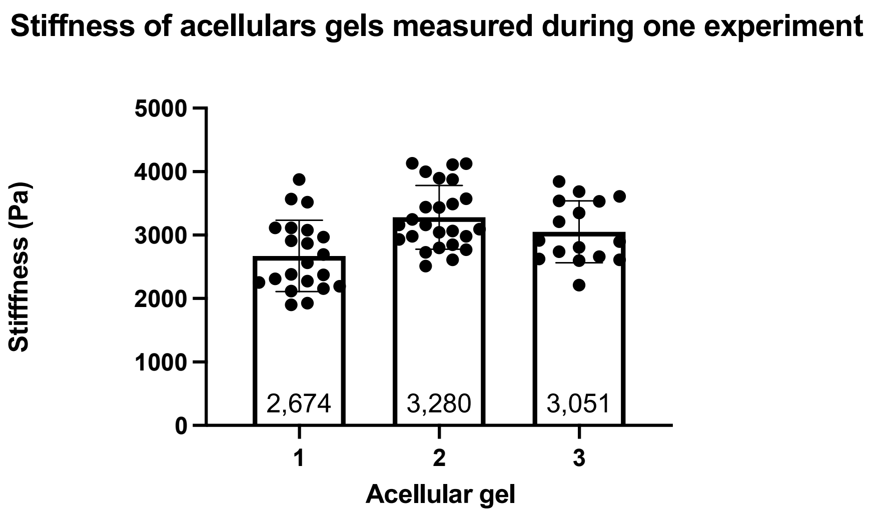

Figure S5 – Reproducibility of the 3D constructs, related to STAR Methods. AFM performed on three different acellular gels, used as controls. n~16 points taken per gel. Data are represented as mean +/- SD.

SUPPLEMENTARY TABLES

Sample ID	G221722800874	G221722801439	G221722801460	G221722801478	G221722801708	G221722801816
**Tumour type**	mucinous adenocarcinoma	adenocarcinoma	adenocarcinoma	adenocarcinoma	adenocarcinoma	adenocarcinoma
**Tumour grade**	moderately differentiated	moderately differentiated	moderately differentiated	poorly differentiated	moderately differentiated	moderately differentiated
**Tumour margin**	infiltrative	pushing	pushing	infiltrative	not stated	infiltrative
**Tumour budding**	not seen	not seen	not seen	present	not stated	present
**Local invasion**	pT4b	pT2	pT3	pT3	pT3	pT3
**Perineural invasion**	No	No	No	Yes	No	Yes
**Lymphovascular invasion**	Yes	No	Yes	Yes	No	Yes
**Number of lymph nodes involved**	3	0	2	1	0	0
**TNM**	pT4b pN1b	pT2 N0	pT3 pN1b	pT3 pN1a	pT3 pN0	pT3 pN0
**Treatment**	Yes	No	No	No	No	No
**Preoperative therapy response**	no evident tumour regression	N/A	N/A	N/A	N/A	N/A
**MMR**	normal	abnormal	normal	normal	normal	normal

Table S1 - Patient information for each CAF sample, related to Figure 1. Compiled by a pathologist.

**Constructs**	**Cell concentration (per gel)**	**Cell concentration (per mL)**
Simple constructs (cancer cells)	270,000	208,000
Simple constructs (CAFs)	135,000	104,000
Simple constructs (CAFs/ cancer cells co-culture)	180,000 cancer cells + 90,000 CAFs	138,000 cancer cells + 69,000 CAFs

Table S2 - Cell concentrations used in the constructs, related to STAR Methods.

	F’ Sequence (5’-3’)	R’ Sequence (5’-3’)
IL6 (BJC Pape 2020[56])	TTGCTGTTATTGTGGTTGTGGTG	CCCATCTCCTTTATCTCAGCCTTC
VIM (AHM Micalet 2022[2])	TCTCTGGCACGTCTTGACCTTG	CGATTTGGACATGCTGTTCCTG
ACTA2 (BJC Pape 2020[56])	CAGGAGGAGAAGGCTGTGTTC	TAAAGGCGGCATCCACTCG
FAP (BJC Pape 2020[56])	CAGTCCACCCTTGTGCTCTTCC	TTCGACTCTCCACGCATCTCTG
PDGFRA (BJC Pape 2020[56])	TACTTCCACAGGTCCCACAACC	GCATTCCTCACAGCCAACAGTG
TGFB1	CACCAACTATTGCTTCAGCTCCAC	TGTCCAGGCAAATGTAGGG
GAPDH (Al Hosni iScience 2022[57])	GCTCTCTGCTCCTCCTGTTC	CGACCAAATCCGTTGACTCC

Table S3 – Primer sequences, related to STAR Methods.

Target	Reference	Dilution
Vimentin	Anti-Vimentin mouse V9 (Santa Cruz, Texas, US)	1:200
Green 2º anti mouse	Anti-mouse Alexa Fluor™ 488 IgG H&L ab150113 (Abcam, Cambridge, UK)	1:1000

Table S4 – Antibodies used for immunofluorescence staining, related to STAR Methods.
